# Muscle fibro-adipogenic progenitors from a single-cell perspective: Focus on their “virtual” secretome

**DOI:** 10.3389/fcell.2022.952041

**Published:** 2022-09-19

**Authors:** Elisa Negroni, Maria Kondili, Laura Muraine, Mona Bensalah, Gillian Sandra Butler-Browne, Vincent Mouly, Anne Bigot, Capucine Trollet

**Affiliations:** Sorbonne Université, Inserm, Institut de Myologie, Centre de Recherche en Myologie, Paris, France

**Keywords:** FAPs, scRNAseq, skeletal muscle, secretome, in silico, extracellular matrix, cell-cell communication

## Abstract

Skeletal muscle is a highly plastic tissue composed of a number of heterogeneous cell populations that, by interacting and communicating with each other, participate to the muscle homeostasis, and orchestrate regeneration and repair in healthy and diseased conditions. Although muscle regeneration relies on the activity of muscle stem cells (MuSCs), many other cellular players such as inflammatory, vascular and tissue-resident mesenchymal cells participate and communicate with MuSCs to sustain the regenerative process. Among them, Fibro-Adipogenic Progenitors (FAPs), a muscle interstitial stromal population, are crucial actors during muscle homeostasis and regeneration, interacting with MuSCs and other cellular players and dynamically producing and remodelling the extra-cellular matrix. Recent emerging single-cell omics technologies have resulted in the dissection of the heterogeneity of each cell populations within skeletal muscle. In this perspective we have reviewed the recent single-cell omics studies with a specific focus on FAPs in mouse and human muscle. More precisely, using the OutCyte prediction tool, we analysed the “virtual” secretome of FAPs, in resting and regenerating conditions, to highlight the potential of RNAseq data for the study of cellular communication.

## Introduction

Skeletal muscle is a highly plastic tissue composed of contractile fibres and a rich connective tissue that accommodates a number of heterogeneous cell populations. Tightly regulated interactions between all these actors regulate muscle homeostasis and support regeneration and repair, in healthy as well as in diseased conditions (Relaix and Zammit, 2012). The extracellular matrix (ECM) is the major constituent of the cellular microenvironment within muscle connective tissue and it is comprised of a large number of components, such as collagens, laminins and fibronectin (Vakonakis and Campbell, 2007; Daley et al., 2008; Hynes and Naba, 2012). In addition to its structural role, the ECM is a dynamic structure that influences many cell functions (e.g., proliferation, migration, adhesion, differentiation, and survival), and participates in vital processes such as angiogenesis, maintenance of the stem cell niche and wound healing (Hynes, 2009; Lu et al., 2011). The ECM is constantly remodelling in response to local or microenvironmental changes through secretion, and modification or degradation of its constituents (Lu et al., 2011). Fibroblasts/stromal progenitor populations are the main producers of ECM not only in the skeletal muscle, but across all organs in the body (Humphrey et al., 2014; de Castro Brás and Frangogiannis, 2020; DeLeon-Pennell et al., 2020; Soliman et al., 2021). Stromal cells/fibroblasts have been described as sentinels of tissue homeostasis, acting as regulators of ECM in order to maintain its mechanical properties. These cells also serve as damage and stress sensors, and respond to injury by promoting tissue regeneration and by mediating the immune response, through secretion of trophic factors and ECM constituents. Nevertheless, when regeneration fails, their action leads to fibrotic infiltration (Caplan and Correa, 2011; Humphrey et al., 2014; Soliman et al., 2021). It is worth pointing out that the nomenclature concerning mesenchymal stem cells/multipotent stromal cells (MSC), activated (myo)fibroblasts and even pericytes/mural cells is somehow confusing and their relationship is not always clear. We invite the reader who wants more information on this subject to refer to these very recent reviews (Contreras et al., 2021; Soliman et al., 2021; Ritso et al., 2022). In this perspective we will focus our attention on stromal non-myogenic cell populations of the adult skeletal muscle that are currently named in the literature as fibro/adipogenic progenitors (FAPs) or fibroblasts. Assuming their pivotal role in ECM secretion, we believe that a major interest should be focused on the *in vivo* secretome of these cells. In order to do this, we extracted the FAPs RNAseq data from publicly available mouse and human skeletal muscle single-cell (sc) data sets (De Micheli et al., 2020b; Oprescu et al., 2020) and subjected these “single-cell FAPs data” to computational filtering to predict the cellular localization and potential secretion of their products. As a prediction tool, we used the OutCyte program which covers largely both conventional (signal-peptide, SP) and unconventional protein secretion (UPS) (Zhao et al., 2019). UPS covers proteins that are secreted without entering the ER–Golgi conventional pathway, usually triggered by cellular stresses such as inflammation or endoplasmic reticulum stress (Rabouille, 2017). We believe that such “virtual” FAPs secretome will give useful information to further decipher cell-cell communication within skeletal muscle.

## Role of FAPs in muscle homeostasis, regeneration and fibrosis

More than 10 years ago, two research groups showed independently in murine skeletal muscle the presence of stromal cells, characterized by the expression of the marker pdgfra (aka CD140a), cd34 and Sca1 (Ly6a/e) (Joe et al., 2010; Uezumi et al., 2010). These cells are localized within the muscle interstitium (endomysium, perimysium and epimysium) and are often observed around vessels (Joe et al., 2010; Uezumi et al., 2010; Contreras et al., 2021; Ritso et al., 2022). Given their dual ability to give rise to adipocytes and fibroblasts both *in vitro* and *in vivo* (Joe et al., 2010; Uezumi et al., 2010; Heredia et al., 2013), they were termed fibro-adipogenic progenitors (FAPs). During homeostasis FAPs are quiescent (Lemos et al., 2015) but they rapidly activate and proliferate in response to muscle damage. As muscle regeneration begins, a finely tuned cross-talk between the different cellular players takes place: immune cells secrete molecules that sustain FAPs proliferation (IL4/IL13) (Heredia et al., 2013), muscle fibres secrete IL15, that stimulates FAPs proliferation and at the same time inhibits their adipogenic differentiation (Kang et al., 2018). FAPs in return, secrete many different molecules creating a favourable environment to support muscle regeneration by allowing interaction directly with muscle fibres/MuSCs and by modulating the activity of other cell populations involved in this process (Heredia et al., 2013; Lemos et al., 2015; Fiore et al., 2016). FAPs produce interleukins/myokines such as IL6 and IL10, known to favour myogenic differentiation (Strle et al., 2007; Serrano et al., 2008; Lemos et al., 2012), and IL33, which induces the proliferation of muscle resident Treg, required for a proper regeneration (Kuswanto et al., 2016). To have a more complete view of the interactions between FAPS, MuSC and immune cells, refer to very recent reviews (Biferali et al., 2019; Contreras et al., 2021; Ritso et al., 2022).

FAPs finally produce many of the ECM proteins, such as proteoglycans, collagens, laminins, fibronectin, to create the proper matrix environment for the ongoing regeneration. The balance between TGFbeta and TNFalpha (both secreted at least by macrophages) allows the proliferation of FAPs and ECM secretion, as well as their clearance by apoptosis in the late phase of regeneration. This clearance is a crucial event to prevent their differentiation toward adipose or fibrogenic phenotype, leading to permanent tissue scarring deposition characterized by an excessive ECM deposition, fatty infiltration (Uezumi et al., 2010; Heredia et al., 2013; Lemos et al., 2015) as well as calcification in some pathological conditions (Mázala et al., 2020). This makes FAPs, together with impaired MuSCs and fibres, one of the actors of the detrimental evolution in pathological condition. Given their anatomical localisation, FAPs are probably heterogeneous even in a steady state, and this heterogeneity is increased during regeneration (De Micheli et al., 2020a), when FAPs engage local cross-talks with different inflammatory cell partners.

## FAPs/fibroblast heterogeneity by RNA omics-approaches

Recently, emerging single-cell omics technologies (sc-RNA-seq, scATACseq, CyTOF) have revealed the *in vivo* heterogeneity of FAPs, in mouse and human, during muscle homeostasis and regeneration. In mouse muscle, FAPs are generally clustered on the basis of Pdgfra (Ly6a/e—coding for Sca1-and Cd34) expression (Dell’Orso et al., 2019; Giordani et al., 2019). In 2019, Scott et al., identified Hic1 (Hypermethylated in cancer 1) as a marker of mesenchymal progenitors in murine skeletal muscle and they showed that the vast majority of Hic1 expressing cells are Pdgfra + Ly6a + Cd34 + FAPs. In resting conditions, they sub-clustered FAPs into two populations (see Table 1 for details): one population expressing genes associated with ECM components and a second one enriched in transcripts involved in cell signalling communication (Scott et al., 2019). Two other papers investigating the single-cell transcriptional profile of FAPs in murine skeletal muscle, suggest that in resting muscles, FAPs can be grouped into two clusters based on the expression of Dpp4 and Cxcl14 (Oprescu et al., 2020) or Lum+ and Fbn1+ (Rubenstein et al., 2020). Other discriminating markers were also identified such as Tie2 and Vcam1 (Malecova et al., 2018), many of them have been detailed in recent reviews (Giuliani et al., 2021a; Contreras et al., 2021; Theret et al., 2021; Ritso et al., 2022). During muscle regeneration, the number of FAPs subpopulations rapidly increases upon injury, with the appearance of different clusters of FAPs (De Micheli et al., 2020a; Oprescu et al., 2020). Although these two studies present differences in the muscle regeneration model (myotoxin injury, volume of myotoxin injected, kinetics of regeneration), both describe a similar behaviour of FAPs during regeneration: 1) rapidly upon injury, FAPs become activated and are transcriptionally different from those in non-injured muscle; 2) at early time points FAPs express genes implicated in cytokine secretion; 3) progressing through later stages they express ECM related genes, implicated in the remodelling of the ECM and indicating resolution of regeneration and return to homeostasis. Interestingly, in the Oprescu et al. study, the 21 days post-injury time-point allowed the identification of both a FAPs population (Osr + FAPs; Odd skipped-related 1) and a fibroblast population (enriched in collagen production). Pseudotime trajectory analysis showed that the Osr + FAPs are at the origin of the two FAPs populations identified in the resting muscle (Dpp4+FAPs and Cxcl14 + FAPs), suggesting that FAPs are in a perpetual state of dynamic adaptation during regeneration (Oprescu et al., 2020).

**TABLE 1:** Sc-RNA-seq unbiased studies showing FAP populations in muscle homeostasis.

References	Species	Markers	FAP (sub)populations	No. of cells in the study	Muscle	Comments
Giordani L et al. (2019)	Mouse	Pdgfra, Ly6a, Ly6e, Dcn	Gsn, Col3a1, Smoc2,Clec3b, Pi16, Lul, Cxcl14, Ugdh, Myoc, Dcn, Serping1, Fstl1	12,441	Adult wt hindlimbs	The single-cell transcriptomics is combined with single-cell mass cytometry (CyTOF; 26 markers, 350k cells)
Dell’Orso S et al. (2019)	Mouse	Pdgfra	Not provided in the paper	4,414	Adult wt hindlimbs	
Oprescu SN et al. (2020)	Mouse	Pdgfra, Cd34, Ly6a	FAP1 (Dpp4+) (Dpp4, Fbn1, Pi16, Wnt2, Igfbp5, Igfbp6, Ugdh, Cd55, Efemp1, Sema3c, Col14a1); FAP2 (Cxcl14+) (Cxcl14, Smoc2,Gsn, Dcn, Apod, Lum, Crispld2, Hsd11b1, Clec3b, Egr1, Col15a1, Egr1)	53,193	Adult wt TA	This paper studies as well the gene expression profile of adult wt mouse TA injured with cardiotoxin (50microl -10microM) at the following time points: n.i., 0.5, 2, 3.5, 5, 10, 21 days post injury
De Micheli AJ et al. (2020a)	Mouse	Pdgfra	FAP (Gsn, Dcn, Col1a1, Col1a2; Col3a1, Lum, Bgn, Smoc2,Cxcl14, Clec3b, Ccl11, Sparc, MyoC, Mfap5, Pi16)	34,438	Adult wt TA	This paper studies as well the gene expression profile of adult wt mouse TA injured with notexin (10microl -10microg/ml) at the following time points: n.i., 2, 5, 7 days post injury
Rubenstein AB et al. (2020)	Mouse	Pdgfra, Cd34	FAP1 (Fbn1+) (Fbn1, Tek, Cd55, Mfap5, Fstl1, Dcn, Col1a1, Col3a1, Col6a1, Col14a1); FAP2 (Lum+) (Lum, Dcn, Cxcl14, Smoc2, Col1a1, Col3a1, Col4a1, Col6a1, Col14a1 Col15a1)	4,000	Adult quadriceps and diaphragm	
Scott RW et al. (2019)	Mouse	Pdgfra, Cd34, Ly6a	FAP1 (Sbsn, Pi16, Efemp1, Anxa3, Sfrp4, Igfbp5, Sema3c, Dpp4, Tgfrb2, Wnt2); FAP2 (Col6a1, 6a2, 6a3, Smoc2, Cxcl14, Col15a1, Crispld2, Lum, GOs2, Sparcle1, Col4a1, Col4a2, Podn)	7,273	Adult TA (Hic1 reporter mouse)	
De Micheli AJ et al. (2020b)	Human	PDGFRα (DCN, GSN)	Fibroblast 1 (FAP1) (COL1A1, COL1A2, SERP4, SERPINE1, CCL2); Fibroblast 2 (FAP2) (PLAC9, THBS4, FBN1, MFAP5, PCOLCE2, FSTL1, IGFBP6, CD55); Fibroblast 3 (FAP3) (ADH1B, ABCA8, MYOC, SMOC2)	22,000	10 different adult donors, diverse anatomical sites	
Rubenstein AB et al. (2020)	Human	PDGFRα, CD34 (COL1A1, COL3A1, COL6A1)	FAP1 (FBN1+) (PRG4, DCN, FBN1, PCOLCE2, CD55, FSTL1, MFAP5, COL14A1); FAP2 (LUM+) (APOD, LUM, DCN, ADH1B, MYOC, SMOC2, CXCL14, COL4A1, COL15A1)	3,479	Adult vastus lateralis (4 samples from the same biopsy)	
Farup J et al. (2021)	Human	PDGFRα, CD34, COL1A1	FAP1 (SEMA3C, FBN1, FSTL1, PRG4, LINC01133, PCOLCE2, IGFBP5); FAP2 (PLA2G2A, CD55, CD248); FAP3 (SFRP2, CCL2, FBLN1, CFH, LUM); FAP4 (MYOC, APOD, PTGDS, COL15A1, SMOC2, COL6A3, MME, IGF1)	5,000 /donor	4 different adult donors (rectus abdominis or gastrocnemius)	One muscle is obtained from type 2 diabetes mellitus patients (age: 67±5 years, BMI:29,1±2,2kg/m2); 3 from adult non type 2 diabetes mellitus patients but with an elevated BMI (age: 71±7 years, BMI:27,8±3,5kg/m2)

BMI: body mass index, n.i.=not injured

Few studies have so far described human FAPs with scRNAseq technology (De Micheli et al., 2020b; Rubenstein et al., 2020; Farup et al., 2021). Rubenstein et al., based on the expression of PDGFRα and CD34, described two subpopulations of FAPs in resting human muscle: LUMICAN+ (LUM+) FAP and FIBRILLIN 1+ (FBN1+) FAP subpopulation (Rubenstein et al., 2020), reminiscent of the two clusters (Lum+ and Fbn1+) identified in mouse sc-RNA-seq datasets. Interestingly, both populations (both in mouse and in human) strongly express collagen types I, III, and VI. The expression of *COL4A1* and *COL15A1* is restricted to LUM + FAPs, while *COL14A1* is expressed in FBN1+ FAPs. To be noted, in mouse, *Col4a1* and *Col15a1* are also restricted to Lum + FAPs, while *Col14a1*, unlike in human, is expressed by both subpopulations. Moreover, TIE2 (Malecova et al., 2018) is not expressed in human FAPs, while its expression is limited to Fbn1+FAPs in mouse. De Micheli et al. (2020b) integrated 22,000 single-cell transcriptomes from 10 adult human donor muscle samples with diverse anatomical locations. They identified three subpopulations of fibroblast-like cells. The first subpopulation expresses high levels of collagen (*COL1A1* and *COL1A2*), *SERP4*, *SERPINE1*, and *CCL2*; the second subpopulation expresses high levels of *FBN1*, and *MFAP5* and *CD55*; finally, the third subpopulation has a gene expression profile similar to the adipocyte cluster, expressing, even if at lower levels, *ADH1B, ABCA8, MYOC, SMOC2*. Recently in 2021 Farup et al. (2021), studying obese and obese/type2 diabetic human skeletal muscle, described the presence of four subpopulations of FAPs whose markers are detailed in Table 1. Moreover, a re-clustering of the four populations enables one to obtain seven new clusters of FAPs, indicating probably an increased heterogeneity in pathological conditions. Interestingly, one of them, characterized by the expression of *CD90* accumulates in Type 2 diabetic skeletal muscle and is associated with enhanced degenerative remodelling of the ECM in those patients.

## Virtual secretome of FAPs

**FIGURE 1:**
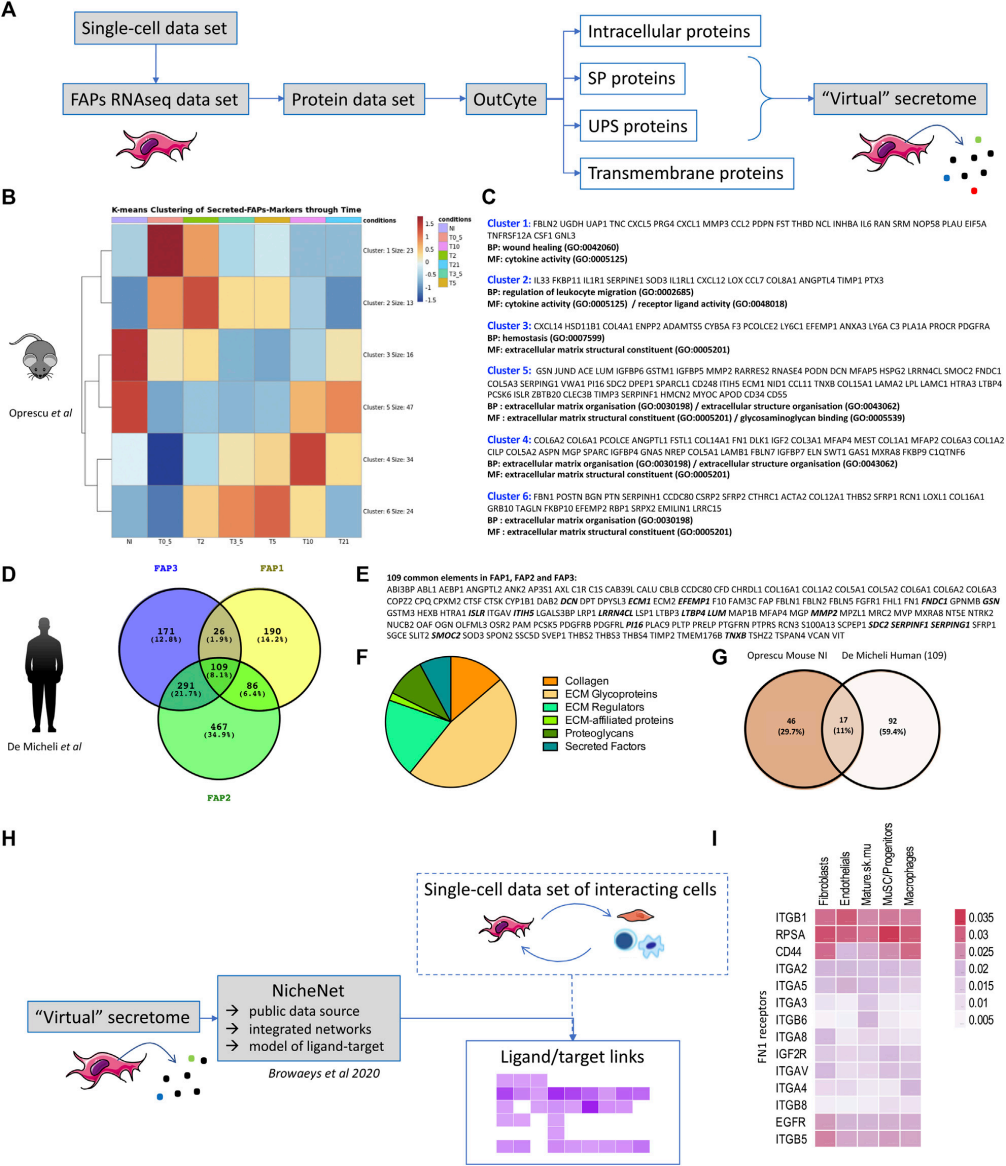
Virtual secretome of murine and human FAPs **(A)** Workflow of the study from sc-RNA-seq datasets to virtual secretome. FAPs RNAseq data-set is an expression matrix that consists of FAPs cells barcodes, as given in each study, in columns and FAPs-markers gene-names in rows. SP = signal-peptide, UPS = unconventional peptide sequence. **(B)** Heatmap of k-means Clustering (with k = 6, produced with pheatmap () function in R) of the genes that are identified as FAPs markers and labelled as secreted (SP,UPS) from the virtual-secretome-tool “Outcyte”. The mean expression of FAPs cells was calculated for each time point per gene. Color values = z-score of expression values. Conditions = NI: non-injured, T0_5 = 0.5 days, T2 = 2 days, T3_5 = 3.5 days, T5 = 5 days, T10 = 10 days, T21 = 21 days after injury. **(C)** Gene names included in each cluster of the heatmap in **(B)**, along with the top-2 Gene Ontology Terms, as given by clusterProfiler package with function “enrichGO ()” and parameters: pvalueCutoff = 0.1, qvalueCutoff = 0.1, pAdjustMethod = “BH”. The enrichment analysis highlights biological processes (BP) and molecular functions (MF) during mouse muscle regeneration. **(D)** Venn Diagram of the marker genes of FAPs subpopulations in human single-cell study, as identified via “FindMarkers ()” function of Seurat package. Values in parentheses are percentages of genes included in each subset. **(E)** List of the common secreted elements/109 genes between the three subpopulations of human FAPs. The proteins highlighted in bold are shared between the two studies. **(F)** Pie chart of human proteins identified as part of the matrisome (ECM and ECM-associated proteins) (Naba et al., 2016). **(G)** Venn diagram presenting the shared proteins between the two studies. The Venn Diagram shows the intersection of gene-names between FAPs-markers highly expressed at the “NI” time point from Oprescu et al and the 109 FAPs-markers in common from the 3 FAPs subpopulations in De Micheli et al. The genes highly expressed in “NI” time point were considered those from clusters 3 and 5 in the k-means clustering in 1B, colored in red. NI: non-injured. **(H)** Workflow of the study to identify downstream receptors and/or downstream regulated gene targets of interacting cells within skeletal muscle. **(I)** Expression of FN1 receptors in several resident cell types within skeletal muscle (extracted from De Micheli et al.).

In order to study the secretory profile of FAPs, we took advantage of two sc-RNA-seq datasets: one published in the study by Oprescu et al, carried out during mouse cardiotoxin-induced regeneration (*GSE138826*; (Oprescu et al., 2020)) and one published by De Micheli et al on human skeletal muscle (*GSE143704*; (De Micheli et al., 2020b)). We opted for the Oprescu et al study because this study covered seven time points providing a good overview of muscle regeneration: from early time points (0.5 days after cardiotoxin injury) till very late time point (21 days) corresponding to a nearly regenerated muscle. We opted for the De Micheli *et al* study, since the authors analysed FAPs from 10 different human muscles, to take into account the heterogeneity between human muscles (Bensalah et al., 2022) and potentially reveal subsets of specific subpopulations of FAPs in specific muscles. For both studies, we subselected the FAPs marker genes and the FAPs annotated-cells to obtain a new dataset of FAPs RNAseq. The FAPs markers were either given in supplementary data (in Table S2 from (Oprescu et al., 2020)), or extracted from function “FindMarkers ()” with Seurat package (Hao et al., 2021). The genes of these RNA-seq data were then translated into peptide-sequences via the biomaRt (Smedley et al., 2009) function “getBM()”, using the latest version of ensembl database (Cunningham et al., 2022) for human (GRCh37) or mouse (GRCm38). The virtual secretome of these protein sequences was obtained using Outcyte (Zhao et al., 2019) as a local standalone version **(** Figure 1A). As mentioned above, our analysis focused on proteins categorized as “signal-peptide” (SP) or “Unconventional protein secretion” (UPS), which are both considered to be secreted extracellularly.

Mouse: Seven time points had been studied in Oprescu et al. (2020), providing a good overview of muscle regeneration: early time points (0.5, 2, 3.5 and 5 days after injury) characterized by inflammatory infiltration and muscle degeneration; an intermediate regeneration stage (5 days) matching with MuSCs proliferation and early differentiation; a later time point (10 days) with a fibre regeneration in progress and finally a very late time point (21 days) corresponding to a terminal phase of muscle regeneration. By clustering of the FAPs marker genes that were predicted as secreted (Figure 1B) we illustrates gene profiles per time point highlighting biological processes (BP) and molecular functions (MF) classically described during muscle regeneration (Figure 1C). According to literature, we found in the non-injured muscle the secretion of proteins like Gsn, Dcn, Lum and Pi16 (cluster 5) already described as produced by FAPs as well as Cxcl14 and Anxa3 (cluster 3) (Giordani et al., 2019; Leinroth et al., 2022). As early as 0.5 days after injury (cluster 1) we found the secretion of proteins like Follistatin (Fst) (Mozzetta et al., 2013), Il6 (Serrano et al., 2008), Cxcl5 and Cxcl1, known to attract monocytes and neutrophils (Soliman et al., 2021) and the HGF-activating enzyme Plau (urokinase plasminogen activator) (Sisson et al., 2009), likely responsible for the activation of satellite cells (Kim et al., 2022) in the first phases of the regeneration. From day 2, we found secreted Il33 that regulates Treg (cluster 2) (Kuswanto et al., 2016) but also the tissue inhibitor of metalloproteinases Timp1, that is described as upregulated by FAPs when cultured in a fibrotic inducing medium (Uezumi et al., 2011) and recently shown to be highly induced in mdx Sca1^high+^FAPs upon culture *in vitro* in a fibrogenic medium (Giuliani et al., 2021b). At day 3.5 and day 5 (cluster 6) FAPs secrete ECM proteins like Fibrillin 1 (Fbn1), Periostin (Post), Biglycan (Bgn), Collagen Triple Helix Repeat Containing 1 (Cthrc1) and Transgelin (Tagln), but also ECM proteins like Periostin (Post), that has been shown to be expressed in the interstitial space at day 5 following a cardiotoxin-induced regeneration and implicated in the maintenance of the muscle mass, suggesting that one possible source of this protein might be FAPs (Ito et al., 2021). Secretion of collagen 6 (col6a1, col6a2, col6a3), starting from day 10 (cluster 4), is also well documented in the literature as produced by fibroblasts (Urciuolo et al., 2013). At day 21, the secretome of FAPs slowly goes back to the muscle homeostasis (cluster 5).

Human: In the De Micheli et al. study, 22,000 single-cell transcriptomes obtained from 10 muscles biopsies originating from different donors were pooled. They grouped fibroblasts in three clusters (here called FAP1, FAP2 and FAP3). While secretome of each cluster would also be of interest (the high number of individual secreted proteins within each group highlights their heterogeneity), here we only looked at the overlap between the list of secreted proteins (SP and UPS) of each FAPs subcategory (FAP1, FAP2 and FAP3), and we identified 109 shared secreted proteins (Figures 1D,E). 47% of these proteins (51/109) were further identified as being part of the matrisome (ECM and ECM-associated proteins) (Naba et al., 2016) with core matrisome proteins (collagens and ECM glycoproteins) and matrisome-associated proteins (ECM regulators, secreted factors, ECM-affiliated proteins) (Figure 1F). We further compared these 109 human secreted proteins to mouse FAPs-secreted proteins identified as highly expressed in cluster 3 and 5 of k-means clustering of Oprescu et al. data (Figure 1B), and thus corresponding to non-injured mouse skeletal muscle. 17 proteins were shared between the two studies (Figure 1G and proteins highlighted in bold in Figure 1E) highlighting common secreted proteins between human and mouse FAPs. To identify downstream receptors and/or signalling pathways in other cell types resident within skeletal muscle, tools such as Nichenet (Browaeys et al., 2020) can be used to exploit public data repositories of ligand-receptor couples and their effect on gene regulation and determine activity and regulation for secreting and receiving cells (Figure 1H). To illustrate this, we took the 109 proteins commonly “virtually secreted” by human FAPs, intersected them with known ligand/receptors network of Nichenet to define a list of ligands and combined that with the list of predicted receptors expressed in several cell-types of interest (fibroblasts, endothelial, mature skeletal muscle, MuSC, macrophages) from the single-cell data set of the De Micheli et al. study. For example, for Fibronectin 1 (FN1) we identified several ligand/receptor couples in each cell type (Figure 1I) that could be further studied to define the downstream targets and pathways of such secreted molecules. Of course, knowing that mRNA levels may not correlate with proteins levels, all these in silico/virtual data require further investigation and confirmation *in vitro* and *in vivo* in “wet” experimental settings.

## Discussion

Muscle regeneration, as other tissue repair, involves many cell types: resident muscle stem cells, endothelial cells, inflammatory cells, and non-inflammatory interstitial cells (pericytes, FAPs). Their complex orchestration during the process of muscle regeneration requires a well-defined and finely-tuned dialogue between all cell types so that each one can play its role at the right time and place. This dialogue is powered through a set of secreted molecules allowing co-regulation through cell to cell communication. Therefore, secretome studies on each cell type are deeply needed to decipher this orchestration. While the secretome of each cell type can be studied individually *in vitro* in culture systems with proteomics approaches (Le Bihan et al., 2012), the validation of these secretomes *in vivo* within skeletal muscle and during regeneration is challenging (Wei et al., 2021). The high-throughput scRNAseq studies of skeletal muscle allow an in-depth analysis of skeletal muscle regulation at cell-type resolution in diverse conditions, and we emphasize in this report the use of a virtual secretome analysis using elaborated bioinformatic tools available (e.g., Outcyte (Zhao et al., 2019) and Nichenet (Browaeys et al., 2020) among others) to add another layer of reading of these single cell analyses. In addition to gene expression, such studies will allow deciphering regulation and signalling pathways within both secreting and receiving cells in physiological and pathological conditions. Altogether we are convinced that in addition to the “wet” experiments, there are a plethora of omics data available as well as databases [e.g., SPRomeDB (Chen et al., 2019)] that need to be fully analysed and merged to have the most complete mapping overview of cell-cell interaction within a given tissue. Virtual secretome is one possibility, that combined with other bioinformatic approaches, will be essential to reveal and decipher these cell communications.

## Data Availability

Publicly available datasets were analyzed in this study. This data can be found here: GSE138826 and GSE143704. The scripts and datasets developed for the analysis are also published at https://github.com/mariakondili/Secretome_in_SingleCell_RNAseq_skeletal_muscle.
